# Long-term outcomes of ductal carcinoma in situ of the breast: a systematic review, meta-analysis and meta-regression analysis

**DOI:** 10.1186/s12885-015-1904-7

**Published:** 2015-11-10

**Authors:** Kirsty E. Stuart, Nehmat Houssami, Richard Taylor, Andrew Hayen, John Boyages

**Affiliations:** Westmead Breast Cancer Institute, Westmead Hospital, PO Box 143, Westmead, NSW 2145 Australia; Crown Princess Mary Cancer Centre, Westmead Hospital, Westmead, Australia; Sydney Medical School, University of Sydney, Sydney, Australia; School of Public Health, Sydney Medical School, University of Sydney, Sydney, Australia; School of Public Health and Community Medicine, Faculty of Medicine, University of New South Wales, Sydney, Australia; Australian School of Advanced Medicine, Macquarie University, Sydney, NSW Australia

**Keywords:** Ductal carcinoma in situ, Meta-analysis, Surgery, Radiotherapy, Biopsy, Tamoxifen, Outcomes, Long-term, Meta-regression analysis, Systematic review

## Abstract

**Background:**

To summarize data on long-term ipsilateral local recurrence (LR) and breast cancer death rate (BCDR) for patients with ductal carcinoma in situ (DCIS) who received different treatments.

**Methods:**

Systematic review and study-level meta-analysis of prospective (*n* = 5) and retrospective (*n* = 21) studies of patients with pure DCIS and with median or mean follow-up time of ≥10 years. Meta-regression was performed to assess and adjust for effects of potential confounders – the average age of women, period of initial treatment, and of bias – follow-up duration on recurrence- and death-rates in each treatment group. LR and BCDR rates by local treatment used were reported. Outside of randomized trials, remaining studies were likely to have tailored patient treatment according to the clinical situation.

**Results:**

Nine thousand four hundred and four DCIS cases in 9391 patients with 10-year follow-up were included. The adjusted meta-regression LR rate for mastectomy was 2.6 % (95 % CI, 0.8–4.5); breast-conserving surgery with radiotherapy (RT), 13.6 % (95 % CI, 9.8–17.4); breast-conserving surgery without RT, 25.5 % (95 % CI, 18.1–32.9); and biopsy-only (residual predominately low-grade DCIS following inadequate excision), 27.8 % (95 % CI, 8.4–47.1).

RT + tamoxifen (TAM) in conservation surgery (CS) patients resulted in lower LR compared to one or no adjuvant treatments: LR rate for CS + RT + TAM, 9.7 %; CS + RT(no TAM), 14.1 %; CS + TAM(no RT), 24.7 %; CS(alone), 25.1 % (linear trend for treatment *P* < 0.0001). Compared to CS + RT + TAM, a significantly higher invasive LR was observed for CS(alone), odds ratio (OR) 2.61 (*P* < 0.0001); CS + TAM(no RT), OR 2.52 (*P* = 0.001); CS + RT(no TAM), OR 1.59 (*P* = 0.022). BCDR was similar for mastectomy, breast-conserving surgery with or without RT (1.3–2.0 %) and non-significantly higher for biopsy-only (2.7 %).

Additionally, the 15-year follow-up was reported where all like-studies had ≥ 15-year data sets; the biopsy-only patients had a meta-analysed total LR rate of 40.2 % and the invasive LR rate was 28.1 %. The biopsy-only patients had a ≥ 15-year BCDR (that included women with metastatic disease) of 17.9 %; the ≥ 15-year BCDR was 55.2 % for those with invasive LR.

**Conclusions:**

More local intervention was associated with greater local control for patients with DCIS at long-term follow-up. For patients undergoing breast-conservation, invasive LR was significantly lower when two rather than one adjuvant treatment modalities were given.

**Electronic supplementary material:**

The online version of this article (doi:10.1186/s12885-015-1904-7) contains supplementary material, which is available to authorized users.

## Background

Ductal carcinoma in situ (DCIS) of the breast is more commonly diagnosed as a result of population-based screening [1]. Various surgical and adjuvant treatments have been extensively investigated for DCIS [2–6], but less is known of long-term outcomes, as recurrence is low, death infrequent and may occur years after the original diagnosis [7, 8]. Our earlier meta-analysis yielded summary ipsilateral local recurrence (LR)-rates of 22.5 % for breast-conserving surgery (BCS), 8.9 % for BCS and radiotherapy (RT), and 1.4 % for mastectomy (Mx), with average follow-ups of 68, 62 and 80 months, respectively [2].

We performed a systematic review, meta-analysis and meta-regression, focusing on studies with long-term outcomes (≥10 years) for DCIS categorized by the extent of local intervention ± tamoxifen (TAM) to assess LR and breast cancer death. We aimed to highlight the natural history of DCIS and guide patient management by determining treatment-related long-term outcomes.

## Methods

This is a systematic review comprising study-level meta-analysis and meta-regression segmented by treatment modality.

### Eligibility criteria

Published studies were systematically identified and assessed for inclusion based on pre-defined eligibility criteria: (1) all patients had pure DCIS, with no evidence of invasion or nodal involvement; (2) had a minimum median or mean follow-up of 10 years, (3) provided descriptions and proportions by surgery-type; (4) ipsilateral LR (breast or chest wall) was a minimum reported outcome; (5) outcome data (LR and breast cancer deaths) were documented in relation to surgery-type, and RT delivery for BCS; and (6) minimum of five eligible patients per study were reported.

### Study selection and data collection

All published studies of any design were considered. No language, publication date or study type restrictions were imposed. On August 31 2013, studies were identified by searching MEDLINE (OVID), Evidence-Based Medicine Reviews databases and hand-searching of references. The search strategy and Preferred Reporting Items for Systematic Reviews and Meta-Analyses methodology are online (Additional file 1). To ensure validity of follow-up data, we sought results as closely related to 10 years (minimum) as information in individual studies allowed (Table 1). When there was more than one publication from an institution or group, the latest study with longest follow-up was used to extract 10-year data. Zero patient overlap was an important goal for this analysis. Detailed information on data inclusion methodology is reported online (Additional file 2).

**Table 1:** Characteristics of eligible studies and patients (*n* = 9404) in ductal carcinoma in situ meta-analysis

				Patient age (years)				Follow-up (years)	
Study and publication year	Collection of patient data	Study design	Country	Mean or median	Range	Diagnosed at ≤ 40 years (%)	Number of eligible cases (Adjusted)^a^	Mean or median	Adjusted^a^
Betsill-1978 [32]	1940–1950	R	US	48.2	34–59	20^c^	8	18^f^	10
Millis-1975 [19]	1948–1968	R	UK	47	39–79	20	16	>15	10
Sanders-2005 [29]	1950–1968	R	US	52^b^	33–80	25	25	31	10
Wanebo-1974 [23]	1953–1972	R	US	53	22–86	NR	14	≥10	10
Sunshine-1985 [17]	1960–1972	R	US	ABO	NR	28^d^	85	>10	>10
Akashi-Tanaka-2000 [20]	1962–1995	R	JP	47^b^	19–92	NR	13	13.4	10
Eusebi-1994 [30]	1964–1976	R	IT	48.6	24–77	24	71	17.5^f^	10
Simpson-1992 [22]	1967–1977	R	US	NR	NR	NR	30	17.7^f^	10
Solin-1996 [27]	1967–1985	R	EU/US	50	26–82	NR	270	10.3	10.3
Lagios-1989 [24]	1972–1980	R	US	54	16–85	NR	20	10.3	10.3
Collins-2005 [31]	1973–1991	R	US	55	39–63	7.7	13	17.4^f^	10
Lara-2003 [21]	1974–1992	R	US	56	31–82	NR	73	19^f^	10
Tunon-de-Lara-2010 [18]	1974–2003	R	FR	36.3	18–40	100	207	13.3	13.3
Di Saverio-2008 [25]	1976–2006	R	IT	ABO	NR	8.5	186	10.8^f^	10.8
Ward-1992 [28]	1979–1983	R	US	58.4^b^	NR	NR	11	>10^f^	10
Shaitelman-2012 [33]	1980–1993	R	US	NR	NR	20.7	145	19.3	10
Ottesen-2000 [13]	1982–1989	P	DK	48^	29–85	NR	168	10	10
Holmes-2011 [34]	1983–2002	R	US	55.5	NR	34^e^	141	10.2	10.2
Fisher-2001 [9] (B-17)	1985–1990	P + RCT	US	ABO	NR	NR	813	10.8	10.8
Vidali-2012 [16]	1985–2000	R	IT	55	29–84	5.5	586	11.3	11.3
Bijker-2006 [10]	1986–1996	P + RCT	EU	53	25–76	6.4	1010	10.5	10.5
Cuzick-2011 [11]	1990–1998	P + RCT	UK/ANZ	ABO	NR	3.3^d^	1694	12.7	12.7
Owen-2013 [14]	1990–1999	P + R	CA	55	27–92	8.6	637	12	12
Wapnir-2011 [12] (B-24)	1991–1994	P + RCT	US	55	NR	17.3	1184	13.6	10
Rudloff-2009 [26]	1991–1995	R	US	55	26–89	15.6^d^	91	11	11
Rakovitch-2013 [15]	1994–2003	R	CA	56	20–85	12.4	1893	10	10

*Abbreviations*: *R* retrospective, *P* prospective, *RCT* randomized controlled trial, *US* United States, *CA* Canada, *UK* United Kingdom, *JP* Japan, *IT* Italy, *EU* Europe, *FR* France, *DK* Denmark, *ANZ* Australia and New Zealand, *ABO* age bands only, *NR* not reported

^a^As close as possible to 10 years from ≥10-year eligible data

^b^for the DCIS patients in study

^c^Included all patients in study

^d^ < 45 years

^e^ < 50 years

^f^mean

Our meta-analysis of recurrence- and death-rates by treatment modality uses study-level data from four prospective RCT trials [9–12], one prospective non-randomized study [13], one study combining prospective and retrospective data [14], and 20 retrospective studies [15–34].

### Data items and endpoints

Information was extracted: (1) study information - number of eligible patients, year published, main author, data-accrual period, institutions involved, length of follow-up and study type; (2) age of patients; (3) treatment modalities - surgery-type, RT, systemic therapy; (4) outcomes (LR and breast cancer deaths).

Primary clinical endpoints for meta-analysis were LR, defined as subsequent ipsilateral breast or chest wall (DCIS or invasive) disease, and breast cancer death rate (BCDR), defined as number of deaths from breast cancer divided by all eligible DCIS cases. The effect of adjuvant therapy in the DCIS breast-conservation population was examined. For treatment groups where *all* studies had mean or median follow-up of ≥15 years (only the biopsy group), further endpoints were examined: the 15-year LR rate and the “≥15 year BCDR” (which included patients with metastatic breast cancer).

Patients included in the biopsy-only group received excision biopsies, with no attention to margins, as the only treatment; these cases, with previously incorrect diagnoses of benign breast disease, were identified as DCIS on retrospective slide review [29–32]. Also, we included data from two BCS trials that documented 3 % of cases with micro-invasive disease: both reported on repeat statistical analysis with the pure DCIS population, and a difference in LR was not detected when compared with the initial cohort [10, 11]. Reasons for exclusion of some patients from eligible trials are outlined online (Additional file 3). Margin analysis was not possible due to lack of margin-specific data.

### Summary measures and statistical analysis

The 95 % confidence intervals (CIs) of LR and BCDR for each individual study treatment-category of Mx, BCS with RT (BCS + RT), BCS without RT (BCS) and biopsy-only were calculated using exact binomial [35], or Poisson [35] for zero numerators.

Meta-analysis combined same-treatment-categories to produce pooled breast cancer-recurrence- and death-rates. A random effects model used an exact likelihood method in which within-study variance was based on binomial distribution [36].

Odds ratios (OR) of LR within the four main treatment groups (Mx, BCS + RT, BCS, biopsy-only) (Table 2), and also adjuvant treatments combinations (±RT,±TAM) in the breast-conservation population (Table 3), were calculated for all treatment groups. Biopsy-only was comparator in the four main treatment groups. BCS without adjuvant treatment, CS(alone), was comparator in the analysis of effect of adjuvant treatment in breast conservation patients.

**Table 2:** Ipsilateral local recurrence and breast cancer death rates in ductal carcinoma in situ by four main treatment groups (Mastectomy, Breast-Conserving Surgery with or without Radiation Therapy, and Biopsy-only) at ten years - meta-analysis and meta-regression

				Meta-analysis^a^				Meta-regression^b^			
								Unadjusted	Adjusted for weighted mean age & period, & 10-year follow-up		
Treatment	Groups	DCIS cases	Local recurrence or death	Rate (%) & 95 % CI	Model	*P* heterog	I^2^ heterog^c^	Rate (%) & 95 % CI	Rate (%) & 95 % CI	Odds ratio	*P*
All local recurrence											
Model *P*-value								*P* < 0.0001	*P* < 0.0001	*P* < 0.0001	
Biopsy	4	117	28	35.8	R	<0.001	83.6	29.8	27.8	14.15	<0.001
				13.4–58.2				15.9–43.8	8.4–47.1	5.26–38.03	
BCS	11	2605	653	25.2	R	<0.001	86.7	23.9	25.5	12.59	<0.001
				19.8–30.6				18.0–29.9	18.1–32.9	6.28–25.26	
BCS + RT	13	5746	716	13.0	R	<0.001	78.4	12.7	13.6	5.79	<0.001
				10.9–15.1				9.6–15.8	9.8–17.4	2.90–11.55	
Mastectomy	8	936	22	3.0	F = R	ns	53.6	2.6	2.6	1.00	
				0.9–5.0				1.0–4.2	0.8–4.50	-	
Total	36	9404	1419								
Linear trend treatment								*P* < 0.001	*P* < 0.001	*P* < 0.001	
Invasive local recurrence											
Model *P*-value								*P* < 0.0001	*P* < 0.0001	*P* < 0.0001	
Biopsy	4	117	21	26.6	R	<0.01	77.1	21.0	10.9	6.83	<0.001
				9.5–43.8				10.4–31.7	3.2–18.5	2.91–16.04	
BCS	11	2601	290	11.0	R	<0.01	64.7	10.6	10.7	6.72	<0.001
				8.6–13.4				7.8–13.4	8.0–13.4	3.83–11.80	
BCS + RT	10	5499	357	7.4	R	<0.001	80.1	6.7	6.7	4.00	<0.001
				5.2–9.5				5.1–8.4	5.4–8.0	2.26–7.08	
Mastectomy	8	853	19	2.5	R	<0.05	58.1	2.1	1.8	1.00	
				0.49–4.5				0.8–3.4	0.8–2.8		
Total	33	9070	687								
Linear trend treatment								*P* < 0.001	*P* < 0.001	*P* < 0.001	
Breast cancer deaths											
Model *P*-value								*P* = 0.3670	*P* = 0.1689	*P* = 0.1689	
Biopsy	4	117	6	5.2	F = R	ns	18.5	4.9	2.7	2.02	0.262
				0.3–10.0				0.5–9.3	0.0–6.0	0.59–6.91	
BCS	9	2296	59	2.6	F = R	ns	0	2.3	2.0	1.5	0.256
				1.9–3.2				1.4–3.3	0.9–3.1	0.75–3.02	
BCS + RT	10	3751	83	2.4	R	<0.001	73.0	2.2	1.9	1.41	0.319
				1.5–3.4				1.5–2.9	1.2–2.6	0.72–2.77	
Mastectomy	8	936	18	2	R	ns	9.1	1.9	1.3	1.00	
				0.9–3.1				0.7–3.1	0.3–2.3	-	
Total	31	7100	166								
Linear trend treatment								*P* = 0.2281	*P* = 0.0685	*P* = 0.0685	

*Abbreviations*: *DCIS* ductal carcinoma in situ, *CI* confidence interval, *BCS* breast-conserving surgery, *BCS* + *RT* BCS + radiation therapy, *F* fixed effects, *R* random effects, *ns* non-significant. Odds ratio *P*-comparator is mastectomy

^a^by variance weighting (Berry)

^b^by non-linear logistic regression of expanded data to unit records

^c^I^2^ assesses heterogeneity of the studies in treatment categories (<30 minimal, 30–75 moderate, >75 considerable)

**Table 3:** Ipsilateral local recurrence and breast cancer death rates in ductal carcinoma in situ breast conservation cases by adjuvant treatment (Conservation Surgery Alone, Conservation Surgery with Radiation Therapy or Tamoxifen, and Conservation Surgery with both Radiation Therapy and Tamoxifen) at ten years - meta-analysis and meta-regression

				Meta-analysis^a^				Meta-regression^b^			
								Unadjusted	Adjusted for weighted mean age & period, & 10-year follow-up		
Treatment	Groups	DCIS cases	Local recurrence or death	Rate (%) & 95%CI	Model	*P* heterog	I^2^ heterog^c^	Rate (%) & 95 % CI	Rate (%) & 95 % CI	OR	*P*
All local recurrence											
Model *P*-value								*P* = 0.0002	*P* = 0.0003	*P* = 0.0003	
CS(alone)	10	2038	541	25.9	R	<0.001	85.5	24.9	25.1	3.12	0.001
				19.9–32.0				19.1–30.6	18.3–31.9	1.62–6.00	
CS + TAM(no RT)	1	567	112	19.8	R	<0.001	86.7	19.7	24.7	3.05	0.024
				16.5-23.0				7.1–32.3	7.9–41.5	1.16–8.04	
CS + RT(no TAM)	11	4809	630	14.9	R	<0.001	78.4	13.8	14.1	1.52	0.190
				11.8-18.0				10.6–16.9	10.3–17.8	0.81–2.86	
CS + RT + TAM	2	937	86	9.2	F = R	ns	53.6	8.5	9.7	1.00	
				7.3-11.0				3.9–13.2	4.4–15.0	-	
Total	24	8351	1369								
Linear trend treatment								*P* < 0.001	*P* < 0.0001	*P* < 0.0001	
Invasive local recurrence											
Model *P*-value								*P* = 0.0005	*P* < 0.0001	*P* < 0.0001	
CS(alone)	10	2038	241	11.4	R	<0.05	60.1	11.2	11.3	2.61	0.001
				8.8–14.1				8.8–13.7	8.9–13.8	1.71–3.97	
CS + TAM(no RT)	1	567	49	8.6	-	-	-	8.6	11.0	2.52	0.001
				6.7–10.6				4.1–13.1	6.2–15.8	1.44–4.41	
CS + RT(no TAM)	8	4562	317	7.7	R	<0.001	79.0	7.4	7.2	1.59	0.022
				5.9–9.5				5.8–8.9	6.1–8.3	1.07–2.35	
CS + RT + TAM	2	937	40	4.3	F = R	ns	<0	4.1	4.7	1.00	
				3.0–5.6				2.2–6.0	3.0–6.4	-	
Total	21	8104	647								
Linear trend treatment								*P* < 0.0001	*P* < 0.0001	*P* < 0.0001	
Breast cancer deaths											
Model *P*-value								*P* = 0.6160	*P* = 0.4152	*P* = 0.4152	
CS(alone)	8	1729	40	2.3	F = R	ns	<0	2.1	2.1	1.15	0.734
				1.6-3.0				1.1–3.1	0.9–3.2	0.52–2.51	
CS + TAM(no RT)	1	567	19	3.4	-	-	-	3.3	4.0	2.24	0.128
				2.1–4.6				0.6–5.9	0.2–7.7	0.79–1.27	
CS + RT(no TAM)	8	2814	67	2.7	R	<0.001	76.4	2.3	2.2	1.21	0.613
				1.4–3.9				1.5–3.2	1.2–3.1	0.58–2.54	
CS + RT + TAM	2	937	16	1.7	R	ns	<0	1.6	1.8	1.00	
				0.9–2.5				0.5–2.8	0.6–3.0	-	
Total	19	6047	142								
Linear trend treatment								*P* = 0.8591	*P* = 0.5586	*P* = 0.5586	

*Abbreviations*: *DCIS* ductal carcinoma in situ, *CI* confidence interval, *CS* conserving surgery, *CS* + *RT* CS + radiation therapy, *TAM* tamoxifen, *F* fixed effects, *R* random effects, *ns* non-significant. Odds ratio *P*-comparator is CS + RT + TAM

^a^by variance weighting (Berry)

^b^by non-linear logistic regression of expanded data to unit records

^c^I^2^ assesses heterogeneity of the studies in treatment categories (<30 minimal, 30–75 moderate, >75 considerable)

Meta-regression was performed to assess and adjust for effects of potential confounders for the following: average age of women, period of initial treatment (as a surrogate for timeframe-related treatment and detection effects), follow-up duration for recurrence and death-rates in each treatment group [37]. The models assessed statistical significance, and adjusted recurrence- and death-rates are provided.

### Bias and confounding

Since this analysis is by treatment category at study-level (aggregate) there may be issues of bias and confounding related to differing study characteristics. A detailed discussion is online (Additional file 2).

## Results

### Study and treatment characteristics

Twenty-six studies, published between 1974 and 2013, were eligible; 15 multi-institutional [9–18, 27–31] and the remainder from single institutions [19–26, 32–34]. Four studies were population-based [13–15, 28] (Table 1). A total of 9404 DCIS cases in 9391 women with treated or untreated DCIS (TisN0M0) between 1940 and 2006 are included in this review by treatment type; 50.0 % of cases (4701/9404) were from RCTs.

Eligible studies reported several surgical interventions for DCIS: BCS (14 studies) [9–13, 15, 16, 24–28, 33, 34]; Mx (4 studies) [14, 20, 22, 23]; both BCS and Mx (4 studies) [17–19, 21]; and biopsy-only (4 studies) [29–32]. There were 36 distinct groups of patients for analysis extracted from the 26 studies, treated by Mx, BCS + RT (all cases and ± TAM), BCS (all cases and ± TAM), or biopsy only, with an average of 1.4 treatment types, hence treatment groups, described per study.

DCIS cases were examined according to local treatment received: Mx (936 cases) (10.0 %) [14, 17–23], BCS + RT (5746 cases) (61.1 %) [9–12, 15, 16, 18, 26–28, 33], BCS (2605 cases) (27.7 %) [9–11, 13, 17–19, 24, 25, 34], and biopsy-only (117 cases) (1.2 %) [29–32]. Most patients (88.8 %) in this analysis had BCS (of whom 68.8 % had RT). The median reported whole-breast RT dose was 50 Gy; 7.1 % of the Mx population received RT.

### Results of individual studies and of pooled analysis

**Fig. 1:**
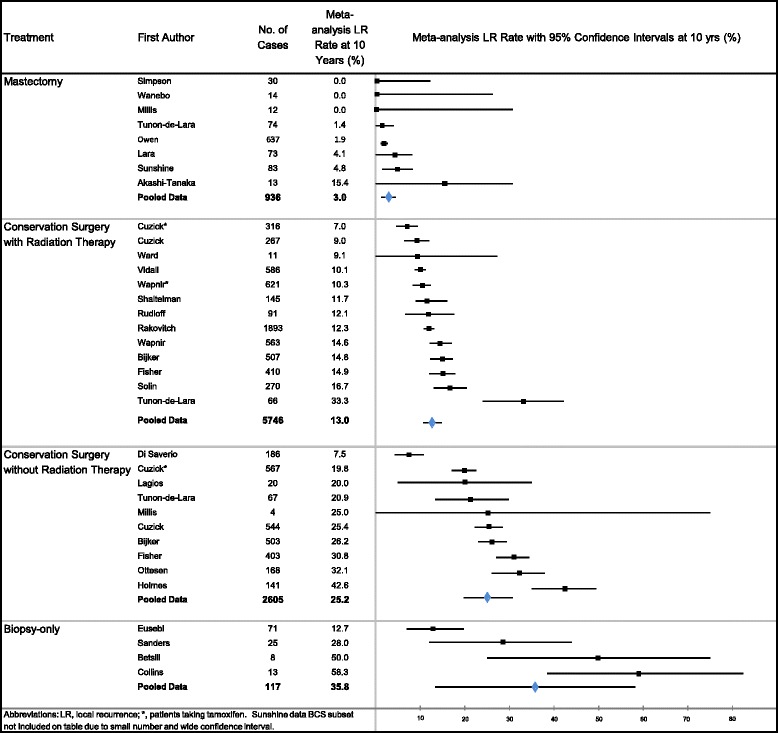
Meta-analysis results: total (invasive and non-invasive) ipsilateral local recurrence rates at 10 years in cases of ductal carcinoma in situ

**Fig. 2:**
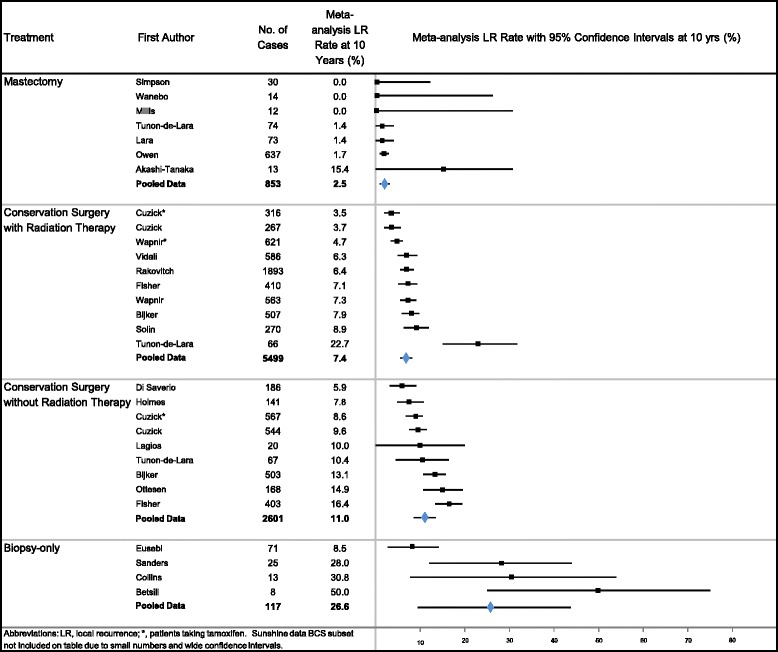
Meta-analysis results: invasive ipsilateral local recurrence rates at 10 years in cases of ductal carcinoma in situ

Table 2 summarizes estimates of LR and BCDR by the four main treatment groups (Mx, BCS + RT, BCS, biopsy-only). The total (invasive and noninvasive) LR rate for Mx was 2.6 %, BCS + RT 13.6 %, BCS 25.5 % and biopsy-only 27.8 %, based on adjusted results from the weighted mean age, period and 10-year follow-up data in the meta-regression. Significant differences in pooled LR-rates on meta-regression analysis were found between Mx and BCS + RT, Mx and BCS, Mx and biopsy-only, and between BCS and BCS + RT (all *P* < 0.0001). Significant differences were seen for invasive LR-rates between Mx and each of the other treatments: BCS + RT, BCS, and biopsy-only; and between BCS + RT and BCS (all *P* < 0.0001). The magnitude of LR-rates for each individual study, and the meta-analyzed summary LR-rates (by treatment category), and their relationship to each other are displayed in Figs. 1 and 2.

Table 3 summarizes estimates of LR and BCDR within the breast conservation population. The addition of both RT and TAM lessened the meta-regression rate of total and invasive LR for patients with DCIS who had conservation surgery (CS), with the lowest total LR rate in patients treated with CS + RT + TAM (9.7 %). Significantly higher rates of total LR occurred in patients treated with CS(alone), 25.1 %; CS + TAM(no RT) 24.7 %; and a non-significantly higher rate was seen in CS + RT(no TAM) 14.1 % (Table 3). A difference was identified between the total LR-rates of CS + RT(no TAM) with CS(alone) (*P* < 0.0001).

Significant differences were seen in the invasive LR meta-regression rates between CS + RT + TAM (4.7 %) and each of the other treatment types: CS(alone) 11.3 %, CS + TAM(no RT) 11.0 %, CS + RT(no TAM) 7.2 %. Significance was noted between the invasive LR OR of CS(alone) compared with CS + RT(no TAM) or CS + RT + TAM, (both *P* <0.0001), but not between CS(alone) and CS + TAM(no RT) (Table 3).

The OR of LR was less with the addition of adjuvant treatment on meta-regression. There was a significant difference between the OR of CS + RT + TAM and the adjuvant treatment groups (CS + TAM(no RT), OR = 3.05; CS(alone),OR = 3.12) for total LR; similar results were observed for invasive LR (CS + TAM(no RT),OR = 2.52; CS(alone),OR = 2.61). Statistical significance was observed for differences in invasive LR between CS + RT + TAM and the adjuvant treatment CS + RT(no TAM) (OR = 1.59). A trend for a higher invasive LR rate was demonstrated for the CS + TAM(no RT) group compared to CS + RT(no TAM) (OR = 1.59, CI 0.99–2.55; *P* = 0.055).

Meta-regression analysis of BCDR at 10 years was similar for the Mx, BCS + RT and BCS patients (1.3–2.0 %), with overlapping 95%CIs. Although the biopsy-only group had a higher BCDR of 2.7 %, this did not statistically differ from estimates for the other three groups (Table 2). A linear trend following the adjusted meta-regression was noted for higher BCDR with less extensive treatment (*P* = 0.0685) (Table 2), but no significance was observed following the adjusted meta-regression for BCDR in any of the combinations of adjuvant therapy compared to patients treated with CS(alone) (Table 3).

Meta-regression adjusting for effects of average age, period of treatment and follow-up duration for total LR and invasive LR produced statistically significant models. While recurrence-rates varied somewhat from the meta-analysis, the relationship remained similar. There were no evident effects of these variables on death-rates, with all models non-significant.

The 15-year follow-up data of biopsy-only patients was examined; the meta-analysed total LR rate was 40.2 % (95 % CI, 17.0–63.4), and the invasive LR rate was 28.1 % (95 % CI, 11.7–44.6). The biopsy-only patients had a ≥15 year BCDR of 17.9 % (95 % CI, 3.8–32.0); the ≥15 year BCDR was 55.2 % (95 % CI, 37.1–73.3) for those with invasive LR.

## Discussion

This overview of long-term (≥10 years) outcomes of 9391 women with 9404 cases of DCIS confirms more extensive local treatment is associated with lower rates of total (DCIS or invasive) or invasive LR. This meta-analysis updates and extends previous work [2], not only with longer follow-up and more studies, but through the additional evaluation of patients who had biopsy-only, adjuvant TAM, and through meta-regression providing adjusted estimates.

Progressively lower estimated proportions of LR are demonstrated with more treatment, from the least radical local treatment (biopsy-only), with the highest LR rate (27.8 %), through BCS (25.5 %) or BCS + RT (13.6 %), to Mx with lowest LR (2.6 %). We found evidence of a reduction in ipsilateral LR (both invasive and DCIS) in those receiving adjuvant RT ± TAM amongst BCS patients. Those who have CS + RT + TAM demonstrate significantly lower invasive LR-rates (4.7 %) than those who receive CS(alone) and only one adjuvant treatment (TAM 11.0 %, RT 7.2 %).

This meta-analysis combines aggregate data from studies to estimate ipsilateral LR and BCDRs in patients with DCIS with more precision than is possible in individual studies. Combining studies increases cases for analysis reducing stochastic variation. Confounding may occur because of differences between studies in treatment categories with respect to age profile, period of diagnosis, and country. Prospective studies are likely to have less measurement bias than retrospective clinical cohort studies. RCTs often have stringent exclusion criteria and often only include a minority of potential cases, whereas observational retrospective studies are more inclusive, providing more generalizable results. Despite the outlined limitations, summarizing published data using meta-analysis may assist clinicians in estimating likely recurrence-rates after various treatments. Outside of RCTs, the remaining studies were likely to have tailored patient treatment according to the clinical situation, with, for example, larger, higher-grade DCIS having more extensive surgery such as a Mx rather than BCS with or without RT. Similar outcomes between different treatment groups treated according to risk does not prove that the treatments are equivalent. This also applies to randomized trials where selected patients with higher risk disease are not offered participation in the trial.

Although this work is based on studies employing various designs and follow-up durations, outcomes data were approximated to 10 years to provide meaningful comparisons. In addition, we investigate outcomes at ≥ 15-years follow-up in the biopsy-only group, finding high rates of LR and breast cancer death; results should be interpreted with knowledge these cases were only identified as having DCIS on retrospective pathology review and may not be entirely representative of outcomes for untreated DCIS. Cases in the biopsy-only group were low-to-intermediate grade DCIS left in situ, and help provide data on the natural history of this disease. Our study highlights even longer periods of follow-up may be necessary to detect survival differences from local therapies, given the average time to ipsilateral invasive breast cancer recurrence for low-intermediate grade DCIS is 131 months and high grade, 76-months [38]. The identified small difference in LR between the biopsy-only and BCS groups could be explained by the possibility positive margins remained after the BCS surgery, the slow natural history of DCIS and that there were only small numbers assessed in the biopsy-only group. Also, DCIS may have been completely excised in the biopsy-only specimens and been less extensive.

Nine hundred and sixty-three DCIS patients treated by Mx are identified with long-term outcomes reported at 10 years–the total LR rate was 2.6 %. This compares with our earlier study with ≤ 10-year follow-up LR rate of 1.4 % (0.7–2.1 %) [2]. Residual subcutaneous and breast tissue containing tumor cells may have been left in situ if suboptimal surgery had been performed, which, in turn, may have raised the LR rate. As results are similar over the years of data collection (1953–2003), with the exception of one paper with small numbers [20], it would appear confounding from differing influences of screening and treatment might not be as great as once thought.

The addition of postoperative irradiation is advantageous for local control for DCIS patients treated with BCS, with adjuvant RT halving LR [2, 3]. Our early meta-analysis recommended the addition of RT to BCS to lower ipsilateral LR risk, particularly in tumors with necrosis, high-grade cytological features, a comedo subtype, or close/positive surgical margins [2]. The DCIS Oxford overview examined data from 3729 women managed with BCS from four RCTs, two of which had 10-year data available [3]. The absolute 10-year risk of any ipsilateral event was reduced from 28 to 13 % with RT; women aged over 50 years had the greatest proportional risk reduction with RT. We include three of these trials in this analysis; our results are in line with those of the Oxford overview. Potentially, this absolute benefit of RT (with minimal toxicity) added to BCS, may be even greater when follow-up time is extended [39]. Long-term SEER data revealed an equivalent left-to-right cardiac mortality ratio when modern RT techniques were used [40, 41]. Scandinavian data detected the rate of major coronary events was proportional to mean heart dose [42]; with improved homogeneity of radiation dosing using 3D and 4D computed tomography planning and intensity-modulated radiation therapy, a decrease in late toxicity is expected [43–45].

Our analysis highlights the effect of adjuvant treatment in lessening total and invasive LR in BCS patients, compared to no adjuvant treatment; those at lowest risk were those who received both adjuvant treatments (RT and TAM). Initial use of TAM in DCIS was sporadic, often without knowledge of hormone receptor status.

The adjusted invasive LR rate in this review did not appear to be lowered with the addition of TAM (CS(alone) 11.3 % versus CS + TAM(no RT) 11.0 %), whereas the addition of RT to CS reduced invasive LR to 7.2 %, dropping further to 4.7 % with CS + RT + TAM. It is puzzling to understand why TAM did not seem to reduce the invasive LR rate; Cuzick et al. reported significant rate reductions with TAM for ipsilateral LR of DCIS (hazard ratio (HR) 0.71), and contralateral LR of invasive (HR 0.47) and DCIS (HR 0.36), but TAM had no effect on ipsilateral invasive LR rates (HR 0.95) [11]. The results suggest that TAM does very little to prevent invasive recurrence on the same side over and above CS alone. We know clinically that elderly patients with invasive breast cancer treated with TAM rather than a mastectomy eventually progress due to tumour resistance to this cytostatic drug [46]. Given the long follow-up in our study, it is possible any residual tumour cells would have become resistant. On the other hand, the ipsilateral LR rate was reduced when TAM was added to CS + RT. RT not only sterilizes residual cancer cells within the breast, but could additionally have a synergistic effect when combined with TAM; whether this is due to post-RT factors within the altered normal breast tissue, or is a weighting of effect of TAM in the prevention of initiation of new cancer.

Wapnir et al. [12] observed an identical 7.5 % 15-year cumulative invasive LR rate in patients with negative margins ± TAM, but positive margins were predictive of TAM response; TAM morbidity of endometrial cancer risk at 163 months doubled in the CS + RT + TAM group (1.7 %) when compared with the CS + RT(no TAM) group (0.78 %) (P = NS). Recent articles estimate DCIS has a mean estrogen receptor rate of 69–79 % and discuss ER as a predictive biomarker for endocrine manipulation [47–49]. A Cochrane review meta-analysis examining the addition of TAM to RT for women with breast conservation for DCIS demonstrated a lower risk of ipsilateral (HR,0.75) and contralateral (HR,0.50) breast events [50]. In two large TAM-RCTs, receptor status was not used in the randomisation process, nor in reporting of outcomes [11, 12]. However, NSABP B-24 has since published evidence of a significant reduction in subsequent breast cancer with TAM use, only when tumor cells were estrogen receptor positive, with no benefit identified in estrogen receptor-negative tumors [51].

Invasive local control results suggesting that patients receiving breast conservation for DCIS may benefit from both RT + TAM must be considered in the context of the risks and benefits of such therapies. Treatment should ideally be discussed in a multidisciplinary forum and include an informed patient’s opinion and consent to validate the final decision. Factors that may influence non-use of TAM include: ER-negative DCIS, patients with a high risk of subsequent complications such as deep venous thrombosis, probability of menopausal symptoms and endometrial cancer (especially age >65 years), particularly when LR-risk is low. Factors in favor of TAM-use may include presence of moderate-to-strong breast cancer family history, significant surrounding atypical hyperplasia or lobular carcinoma in situ, and the BCS + RT patient’s perceived benefit of reducing 10-year risk of invasive LR. The risk of metastatic breast cancer following invasive recurrence from DCIS has been reported as 13–40 % [39, 52, 53]. Breast cancer-specific survival in women diagnosed with DCIS is significantly reduced following an invasive LR; both Donker et al. and Wapnir et al. documented respectively a 17-fold and 7-fold increase in the risk of breast cancer death after an invasive LR compared with those who had a DCIS LR or no LR [12, 53].

No significant difference in BCDR was observed at 10 years between the Mx, BCS + RT and BCS groups, possibly due to early detection and management of recurrences. The biopsy-only group had the highest BCDR at 10 years, and although not statistically different from other groups, may be due to a small number of cases. When ≥15-year follow-up data were used, the ≥15-year BCDR in the biopsy-only group was high (17.9 %), potentially a reflection of latent progression of invasive recurrences to metastatic disease and death. The 10-year BCDRs were no different in patients who had CS ± adjuvant therapy after meta-regression analysis. However, it may be data are not yet mature enough to produce statistical significance, considering the association between reduction in invasive LR and improvement in breast cancer survival [54, 55]. The DCIS Oxford overview did not report a breast cancer survival advantage, but only two of four trials had long-term data [3].

In the NSABP B-17/B-24 studies, cumulative probability of breast cancer-related death was 10.4 %, 10 years after the occurrence of ipsilateral invasive breast cancer recurrence [12]. In EORTC data, patients with ipsilateral invasive LR had a significantly worse breast cancer-specific survival at 60 % (HR,17.66) and overall survival (HR,5.17) ten years after LR, compared to those who had ipsilateral DCIS LR or did not experience a recurrence, with breast cancer-specific survival around 95 % (*P* < 0.001) highlighting that treatment strategy minimizing invasive recurrence is important for some patients [53].

Nomograms can estimate for risk recurrence for women with DCIS. The Van Nuys Prognostic Index and its variations (for Mx and BCS patients) has been evaluated for risk recurrence in independent populations [56], as has the Memorial Sloan-Kettering Cancer Center (MSKCC) DCIS Nomogram (for BCS patients) [57]; the MSKCC data were based on 1681 consecutive women.

Ongoing DCIS management trials include assessment of the role of TAM versus aromatase inhibitors [58] and of the role of trastuzumab in HER2-positive DCIS patients [59]. A validation study of genetic profiling for DCIS recurrence risk is under way [60]. These or other approaches, alone or in combination, may provide an outcome advantage over current management.

## Conclusions

We systematically meta-analyzed DCIS case-data on LR and the BCDR to provide comprehensive summary information on long-term outcomes, accounting for study-level potential confounders.

We have identified that more intensive local intervention was significantly associated with greater local control for patients with DCIS at long-term follow-up. For patients undergoing breast-conservation, invasive LR was significantly lower when two rather than one adjuvant treatment modalities were given. Residual predominately low-grade DCIS following inadequate excision (represented by the biopsy-only group) resulted in high LR and BCDRs at 15 years.

### Ethics

As this is a systematic review and analysis of previously published literature, ethics is not required.

### Standards of reporting

PRISMA methodology has been adhered to in this manuscript and the Additional files. In order to enhance readability of this manuscript, duplication of reported data (as required by PRISMA protocol) was condensed on occasion from two (or several) sections to one section.

### Availability of supporting data

As this is a systematic review, meta-analysis and meta-regression analysis, all eligible papers in the systematic review and analysed are listed in the reference list, and have been clearly listed in the manuscript.

## Additional files

Additional file 1:
**Systematic review flow diagram (adapted from PRISMA recommendations), and information sources and search strategy.** (DOCX 33 kb) Additional file 2:
**Data inclusion methodology, definitions of ipsilateral local recurrence, detailed discussion of bias and confounding factors.** (DOCX 28 kb) Additional file 3:
**Reasons for exclusion of some patients from eligible DCIS studies.** (DOCX 15 kb)

## Abbreviations

BCDR: Breast cancer death rate
BCS: Breast-conserving surgery
CI: Confidence interval
CS: Conservation surgery
DCIS: Ductal carcinoma in situ
HR: Hazard ratio
LR: Ipsilateral local recurrence
MSKCC: Memorial Sloan-Kettering Cancer Center
Mx: Mastectomy
OR: Odds ratio
RT: Radiation therapy
TAM: Tamoxifen
