# Supplementary material for: Long-term outcomes of ductal carcinoma in situ of the breast: a systematic review, meta-analysis and meta-regression analysis
Source: BMC Cancer. 2015 Nov 10;15:890. doi: 10.1186/s12885-015-1904-7 (PMC4641372; doi:10.1186/s12885-015-1904-7)
Supplement: Additional file 1: — Systematic review flow diagram (adapted from PRISMA recommendations), and information sources and search strategy. (DOCX 33 kb) [file 12885_2015_1904_MOESM1_ESM.docx]

**Additional file 1**

**Systematic Review Flow Diagram^1^ (adapted from ^a^ PRISMA recommendations)**

Articles were identified by an electronic search (1965 to 31 August 2013). MEDLINE database was searched by combining the following: (a) DCIS or ductal carcinoma in situ (all subject headings) as a term in the Title (ti.) or the Abstract (ab.) or Keyword Heading Word (kf.); and (b) ‘outcome$, or follow-up, or followup, or follow up, or long term (all subject headings) as terms ti., ab. or kf. The Evidence Based Medicine (EBM) review databases were also searched (1965 to 31 August 2013): the Cochrane Central Register of Controlled Trials was searched with the above methodology; and the other EBM databases were searched with DCIS or ductal carcinoma in situ (all subject headings); all fields for: Database of Abstracts and Reviews of Effects, NHS Economic Evaluation Database, and Health Technology Assessment; the remainder were searched more specifically with ti., ab. or kf.

Articles excluded based on eligibility criteria (n=302):

- Follow-up < 10 years (n=178)
- Follow-up uncertain (n=23)
- No ipsilateral local recurrence follow-up data (n=26)
- No link between patients or their treatment and outcome (n=5)
- Invasive or mixed pathology and/or unable to separate out DCIS data (n=27)
- Review paper only (n=18)
- Other: (n=13) [pathology only (5), imaging only (2), DCIS cost modeling (3), DCIS prevention (1), LCIS only (1), benign only (1)]
- DCIS salvage treatment only (n=2)
- <5 eligible patients in study (n=2)
- Articles listed more than once (n=7)

NB: 1 unable to be found (published 1934)

Potentially relevant full-text articles checked for eligibility (n=131)

^b^ **Additional** hand-checked abstracts and articles screened and checked for eligibility (n=209)

Abstracts of potential relevance screened

(MEDLINE: n=877)

(EBM reviews: n=71)

Articles excluded if irrelevant, clearly not eligible based on abstracts (n=608)

Avoidance of overlapping or duplicate cohorts or subjects (eligible articles reporting data on the same cohort were identified to avoid double-counting subjects): updated: (n=3) [Page (‘82 & ‘95), Eusebi (‘89)]; cohorts may overlap (n=2) [Mirza (‘00), Haffty (‘97)]; same patient outcomes reported: (n=5) [Rudloff (‘10), Ottesen (‘03), Witkiewicz (‘09), Allred (‘12), Wilkinson (‘12)]; specific individual patient outcomes less clear in subsequent paper : (2) [Rosen (‘80), Tunon-de-Lara (’11)].

Potentially eligible full-text articles reviewed to identify possible cohort overlap and for data extraction (n=38)

Primary studies in the systematic review including quantitative synthesis

(meta-analysis and meta-regression analysis) (n=26)

^a^ PRISMA: Preferred Reporting Items for Systematic Reviews and Meta-Analyses

^b^ Additional articles were iteratively identified by checking the references of the eligible primary studies, relevant review articles and other studies identified.

**Information Sources and Search**

Studies were identified by searching electronic databases and hand-searching reference lists of eligible primary studies and many non-eligible studies. Non-English language articles identified by the abstract thought to be potentially eligible, (Ovid Medline abstracts are in English), were translated to assess eligibility.

This search was applied to MEDLINE (OVID) (1965–Present), and adapted for the Evidence-Based Medicine (EBM) review databases (1965–Present): [Cochrane Database of Systematic Reviews](http://ezproxy.library.usyd.edu.au/login?url=http://gateway.ovid.com/ovidweb.cgi?T=JS&MODE=ovid&PAGE=main&NEWS=N&D=coch&PAGE=main); [ACP Journal Club](http://ezproxy.library.usyd.edu.au/login?url=http://gateway.ovid.com/ovidweb.cgi?T=JS&MODE=ovid&PAGE=main&NEWS=N&D=acp&PAGE=main); [Database of Abstracts of Reviews of Effects (DARE)](http://ezproxy.library.usyd.edu.au/login?url=http://gateway.ovid.com/ovidweb.cgi?T=JS&MODE=ovid&PAGE=main&NEWS=N&D=dare&PAGE=main); [Cochrane Central Register of Controlled Trials (CCTR)](http://ezproxy.library.usyd.edu.au/login?url=http://gateway.ovid.com/ovidweb.cgi?T=JS&MODE=ovid&PAGE=main&NEWS=N&D=cctr); [NHS Economic Evaluation Database (CLEED)](http://ezproxy.library.usyd.edu.au/login?url=http://gateway.ovid.com/ovidweb.cgi?T=JS&MODE=ovid&PAGE=main&NEWS=N&D=cleed); [Health Technology Assessment (CLHTA)](http://ezproxy.library.usyd.edu.au/login?url=http://gateway.ovid.com/ovidweb.cgi?T=JS&MODE=ovid&PAGE=main&NEWS=N&D=clhta); [Cochrane Methodology Register (CLMCR)](http://ezproxy.library.usyd.edu.au/login?url=http://gateway.ovid.com/ovidweb.cgi?T=JS&MODE=ovid&NEWS=N&D=clcmr&PAGE=main). The search was performed on 31 August 2013 by K.S.

We used the following search terms to search the MEDLINE and CCTR: (a) DCIS or ductal carcinoma in situ (all subject headings) as a term in the Title (ti.), abstract (ab.), or Keyword Heading Word (kf.); *and* (b) outcome$, long term, follow-up, followup or follow up (all subject headings) as terms ti., ab. or kf. The remaining EBM databases were searched with the terms DCIS or ductal carcinoma in situ (all subject headings). All fields for: DARE, CLEED, and CLHTA; the remainder were searched more specifically with ti., ab. or kf..

Screening and assessment of study eligibility were performed in a standardized manner by one author (K.S.). Articles that could not be excluded or could not be assessed based on title or abstract alone were screened using the full-text article. To identify additional articles, we reviewed the references of all eligible primary studies, relevant review articles and other articles. One author (K.S.) extracted all data items, and a second author (N.H.) was consulted to check extracted data.

Discussion with another author (N.H.) was used to reach consensus about eligibility in instances of uncertainty concerning studies meeting inclusion criteria. A data extraction sheet was developed and pilot-tested on four studies and refined accordingly. Some articles reported on both eligible and non-eligible patients; if the eligible patients’ data were separable, the study was included. Case reports of male DCIS patients were identified, but did not meet eligibility criteria; no males were identified in the 26 eligible studies.

To avoid double counting data, we cross-referenced all author names and institutions identified in eligible studies, and checked time periods of studies for which overlap was possible. Where two or more papers reported data for the same cohort, the most recent paper was preferentially used in this review. Authors were contacted if uncertainly remained (Shaitelman et al. and Holmes et al. were contacted; no patients were involved in the NSABP B-17 or -24 studies). Overlap in this systematic review was estimated as zero.

A study-level minimum mean or median of 10 years’ follow-up was a criterion for study inclusion, and hence some individual patients will have had less (or more) than 10 years of follow-up.

1. Liberati A, Altman DG, Tetzlaff J, Mulrow C, Gotzsche PC, Ioannidis JP, et al. The PRISMA statement for reporting systematic reviews and meta-analyses of studies that evaluate health care interventions: explanation and elaboration. *PLoS Medicine / Public Library of Science* 2009;6(7):e1000100.
