# Supplementary material for: Long-term outcomes of ductal carcinoma in situ of the breast: a systematic review, meta-analysis and meta-regression analysis
Source: BMC Cancer. 2015 Nov 10;15:890. doi: 10.1186/s12885-015-1904-7 (PMC4641372; doi:10.1186/s12885-015-1904-7)
Supplement: Additional file 2: — Data inclusion methodology, definitions of ipsilateral local recurrence, detailed discussion of bias and confounding factors. (DOCX 28 kb) [file 12885_2015_1904_MOESM2_ESM.docx]

**Additional file 2**

**Data inclusion methodology**

**To ensure validity of the 10-year follow-up data, outcome results were related as closely to 10 years as information in individual studies allowed** (Manuscript - Table 1). When there was more than one publication from an institution or group, the latest study with the longest follow-up was used to extract 10-year data. If >10-year data were only available, the results reporting on outcomes closest to a minimum of 10 years’ follow-up were used (e.g. 13-year data were used if 10-year data were not available). If 10-year (or just greater than 10-year) data were not able to be extracted from the latest publication, the previous publication was utilised [e.g. if 15-year+ data were available and 10-year data were not, the previous publication (relating to the 15-year+ study) reporting 10-year data were used].

Some treatment arms in studies not explicitly including tamoxifen in this meta-analysis include a small proportion of patients who did receive tamoxifen.^1-5^ Very few patients received alternative endocrine treatment and, for the purpose of this meta-analysis, they were grouped with those who received tamoxifen. Authors of eligible studies were not contacted for individual patient data.

**Definitions of ipsilateral local recurrence**

|  |  |
| --- | --- |
| **Study and Publication Year** | **Ipsilateral Local Recurrence Description / Definition** |
| Betsill-1978 | Clinical description of each individual patient's data and recurrence within ipsilateral breast/chest wall |
| Millis-1975 | Clinical description of each individual patient's data and recurrence within ipsilateral breast/chest wall |
| Sanders-2005^a^ | Clinical description of each individual patient's data and recurrence within ipsilateral breast/chest wall |
| Wanebo-1974 | No official description of local recurrence; however, all eligible patients “remained free of local recurrence”. Other reported disease recurrence terminology included: metastatic disease and death. |
| Sunshine-1985 | Clinical information of patients with recurrences in the ipsilateral breast/chest wall |
| Akashi-Tanaka-2000 | Clinical information of patients with recurrences in the ipsilateral breast/chest wall |
| Eusebi-1994 | Clinical description of each individual patient's data and recurrence within ipsilateral breast/chest wall |
| Simpson-1992 | All eligible patients did not have a local recurrence |
| Solin-1996 | "Local failure was scored for a failure that occurred within the treated breast" |
| Lagios-1989 | "All recurrences regardless of type were in the ipsilateral breast…" |
| Collins-2005 | "All 10 breast carcinoma events (100%) were in the ipsilateral breast". |
| Lara-2003 | Clinical information of patients with recurrences in the ipsilateral breast/chest wall |
| Tunon-de-Lara-2010 | "Ipsilateral local recurrence was defined as the recurrence of an in situ or invasive cancer in the treated breast" |
| Di Saverio-2008 | Inference: Local recurrence in the context of VNPI (for ipsilateral scoring of risk of recurrence) |
| Ward-1992 | Recurrences described as ipsilateral or contralateral; ipsilateral recurrences were recorded for our meta-analysis. |
| Shaitelman-2012 | "An IBTR was defined as the reappearance of cancer in the treated breast, alone or at the time of distant metastases" |
| Ottesen-2000 | "Recurrence was defined as the subsequent development of either DCIS or invasive carcinoma after the completion of the primary surgical treatment". Ipsilateral recurrences were clearly shown in text and on a table. |
| Holmes-2011 | Local recurrence: "DCIS occurring within the same quadrant of the same breast" or invasive breast cancer |
| Fisher-2001(B-17) | ipsilateral invasive or non-invasive breast cancer |
| Vidali-2012 | Ipsilateral invasive or non-invasive breast cancer in the breast/chest wall |
| Bijker-2006 | "The primary end points were both invasive and DCIS LR in the treated breast". |
| Cuzick-2011 | Ipsilateral invasive or non-invasive breast cancer in the breast/chest wall |
| Owen-2013 | Clinical information of patients with recurrences in the ipsilateral breast/chest wall |
| Wapnir-2011(B-24) | ipsilateral invasive or non-invasive breast cancer |
| Rudloff-2009 | "any subsequent DCIS or infiltrating carcinoma within the treated breast" |
| Rakovitch-2013 | "Any local recurrence [was the] detection of DCIS or invasive breast cancer that developed in the same breast ≥12 months after the initial diagnosis of DCIS." |
| ^a^Previous studies were used to gather information on the ipsilateral local recurrences on each of the women involved | |

**Detailed discussion of bias and confounding factors**

Since this analysis is by treatment category at study-level (aggregate) there may be issues of bias and confounding related to differing study characteristics. Differences in age profile of patients in each treatment subgroup may cause confounding by age. Follow-up data can also be affected by measurement bias; differing length of follow-up was minimised by selecting only cases with a 10-year follow-up, or minimum approximate 10-year follow-up - some studies report mean or median follow-up of 11-13 years. Measurement bias could also occur because of the varying use of mean or median to summarise age and duration of follow-up.

In view of the extended period during which studies were implemented, the period of diagnosis and initial treatment could be a proxy for confounding by the quality of technique and implementation of treatment and early diagnosis from mammography screening (lead-time bias). Furthermore, the country in which studies were carried out may be a proxy for the local epidemiology of breast cancer and the health system under which screening, diagnosis, and treatment took place, amongst other background confounders. Differences in study type are mainly between retrospective longitudinal case-series (clinical cohorts) and prospective studies which were mainly RCTs. There may be less measurement error across a range of variables in prospective studies compared with retrospective cohorts. Age, period of diagnosis and length of follow-up are adjusted for using meta-regression and represents three variables from 36 treatment groups analysed.

**References**

1. Di Saverio S, Catena F, Santini D, Ansaloni L, Fogacci T, Mignani S, et al. 259 Patients with DCIS of the breast applying USC/Van Nuys prognostic index: a retrospective review with long term follow up. *Breast Cancer Research & Treatment* 2008;109(3):405-16.

2. Solin LJ, Kurtz J, Fourquet A, Amalric R, Recht A, Bornstein BA, et al. Fifteen-year results of breast-conserving surgery and definitive breast irradiation for the treatment of ductal carcinoma in situ of the breast. *Journal of Clinical Oncology* 1996;14(3):754-63.

3. Rudloff U, Brogi E, Brockway JP, Goldberg JI, Cranor M, Wynveen CA, et al. Concurrent lobular neoplasia increases the risk of ipsilateral breast cancer recurrence in patients with ductal carcinoma in situ treated with breast-conserving therapy. *Cancer* 2009;115(6):1203-14.

4. Shaitelman SF, Wilkinson JB, Kestin LL, Ye H, Goldstein NS, Martinez AA, et al. Long-term outcome in patients with ductal carcinoma in situ treated with breast-conserving therapy: implications for optimal follow-up strategies. *International Journal of Radiation Oncology, Biology, Physics* 2012;83(3):e305-12.

5. Rakovitch E, Narod SA, Nofech-Moses S, Hanna W, Thiruchelvam D, Saskin R, et al. Impact of boost radiation in the treatment of ductal carcinoma in situ: a population-based analysis. *International Journal of Radiation Oncology, Biology, Physics* 2013;86(3):491-7.
