# Supplementary material for: Long-term outcomes of ductal carcinoma in situ of the breast: a systematic review, meta-analysis and meta-regression analysis
Source: BMC Cancer. 2015 Nov 10;15:890. doi: 10.1186/s12885-015-1904-7 (PMC4641372; doi:10.1186/s12885-015-1904-7)
Supplement: Additional file 3: — Reasons for exclusion of some patients from eligible DCIS studies. (DOCX 15 kb) [file 12885_2015_1904_MOESM3_ESM.docx]

**Additional file 3**

**Reasons for Exclusion of some Patients from Eligible DCIS Studies**

| Study | Treatment | Reason for exclusion |
| --- | --- | --- |
| Lara 2003 | Mx | Pts with micro-metastases were excluded, as were the lumpectomy pts (it was unclear as to whether RT had been delivered or not). |
| Millis 1975 | Mx | 12 Mx pts had been followed-up for ≥ 10 years; others were therefore excluded. |
| Simpson 1992 | Mx | 1 pt who died of breast cancer was not included as she had had a contralateral invasive breast cancer prior to the DCIS diagnosis. |
| Wanebo 1974 | Mx | DCIS pts with < 10 year follow-up were excluded. |
| Rudloff 2009 | CS/RT | CS-only pts’ median follow-up was 7.8 yrs; therefore they were not included. |
| Ward 1992 | CS/RT | CS+RT pt data was included. Mx pt data was excluded as outcomes were not clearly associated with the DCIS only group versus the DCIS and invasive cancer group. |
| Lagios 1989 | CS | 59 of the 79 pts did not have long-term follow-up and were excluded. |
| Millis 1975 | CS | 4 CS pts had been followed up for ≥ 10 years; others were excluded. |
| Betsill 1978 | Bx | 8 pts had 10-year outcome data that was able to be included. |
| Eusebi 1994 | Bx | 71 cases of DCIS in 70 patients were included. 10 patients died of unrelated causes < 10 years from initial biopsy and were excluded. |
| Di Saverio 2008 | CS/RT | 73 pts had CS+RT and were not included as they may have overlapped with Vidali *et al*. The other 168 patients treated with CS only were included in analysis. |
| Abbreviations: DCIS, ductal carcinoma in situ; Mx, mastectomy; pts, patients; RT, radiation therapy; CS+RT, breast-conserving surgery and radiation therapy; CS, breast-conserving surgery without radiation therapy; Bx, biopsy alone. | | |
